# Nutritional Description of Organic and Conventional Food Products in Spain: The BADALI Project

**DOI:** 10.3390/nu15081876

**Published:** 2023-04-13

**Authors:** Ana B. Ropero, Fernando Borrás, Marta Rodríguez, Marta Beltrá

**Affiliations:** 1 Institute of Bioengineering, Miguel Hernández University, 03202 Elche, Spain; beltra@umh.es; 2 Department of Statistics, Mathematics and Informatics, Miguel Hernández University, 03202 Elche, Spain; f.borras@umh.es; 3 Polytechnic School of Orihuela, Miguel Hernández University, 03202 Elche, Spain; marta.rodriguez11@goumh.umh.es

**Keywords:** nutrient composition, organic, nutrient profile/profiling model, nutrition claims, fortification, sweeteners, healthy food, food database, cereal-based products, dairy substitutes

## Abstract

Organic food and drink is undoubtedly a growing market. Consumers perceive organic food as healthy, and nutrition claims (NCs) and fortification may add to this perception. Whether this is true is still a matter of controversy, particularly for organic food products. We present here the first comprehensive study of large samples of six specific organic food types, analysing the nutritional quality (nutrient composition and “healthiness”) as well as the use of NCs and fortification. In parallel, a comparison with conventional food is also carried out. For this purpose, the Food Database of products in the Spanish market, BADALI, was used. Four cereal-based and two dairy-substitute food types were analysed. Our results show that as many as 81% of organic foods are considered “less healthy” by the Pan American Health Organization Nutrient Profile Model (PAHO-NPM). Organic foods present a slightly improved nutrient profile compared to conventional foods. However, many of the differences, though statistically significant, are nutritionally irrelevant. Organic foods use NCs very frequently, more than conventional foods, with very little micronutrient fortification. The main conclusion of this work is that consumers’ perception that organic food products are healthy is unfounded from a nutritional point of view.

## 1. Introduction

The organic food and drink market has increased in recent years, and it is expected to continue growing. According to the latest released data for 2022, it reached 120.6 billion euros in 2020, with the United States as the leading market. In Europe, the growth rate was the highest in the last decade (14.9%) [1,2]. This tendency will continue as the global organic food and drink market is projected to register a mean annual growth rate of 16.5 during 2023–2028 [3]. The number of global organic producers and farmland also increased by 7.6% and 4.1%, respectively, from 2019 to 2020. Cereals and dry pulses constituted the main crops on organic arable land in 2020. As for Spain, it was the second-highest European country in terms of organic farmland [2,4].

Consumers choose organic food for several reasons, including animal welfare and environmental concerns [5]. Health is also one of the main motives, either because of reduced exposure to contaminants or increased nutritional value [5]. In fact, studies suggest that consumers perceive organic food as healthy, even healthier than conventional food [6,7,8]. Consumers consider reducing cancer risk as one of the health benefits attributed to organic foods [9]. They also believe this food to be lower in fat and calorie content as well as higher in fibre [8,10,11]. However, the perceived healthfulness of organic processed foods is diminished compared to whole foods [8].

Nutrition/health claims and nutrient fortification are two factors influencing the perceived healthiness of foods or making them more appealing to consumers [12,13,14,15,16,17]. There is a consensus around the effects of nutrition and health claims. They may increase the perceived nutritional quality and healthiness of products as well as influence purchasing behaviour [14,15,16,17]. Particularly, organic consumers with a high health attitude attribute high importance to nutrition claims (NCs) [18]. Therefore, manufacturers may use NCs and nutrient fortification to potentiate the consumers’ perceptions of these foods as healthy.

Despite consumers’ beliefs, the association between organic food and health benefits is largely uncertain. Some studies have shown positive outcomes of organic food on infertility, birth defects, allergic sensitization, otitis media, pre-eclampsia, the metabolic syndrome, overweight/obesity, lymphoma, postmenopausal breast cancer, and type 2 diabetes [5,19,20,21,22,23]. However, these associations do not imply a causal relationship [5]. In fact, short-term clinical trials have shown no benefit, and long-term trials are lacking [20,21]. Furthermore, lifestyle is likely to be an important confounder. Indeed, it has been reported that organic consumers tend to have healthier lifestyles and dietary patterns, a lower body mass index, and are more physically active [5,19,21,22]. Moreover, there are more vegans and vegetarians among organic consumers [5,21].

The absence of pesticides and the decrease in heavy metals may be the main reasons for the possible health effects of organic food [21]. The contribution of a hypothetical nutritional improvement seems secondary. Studies have shown contradictory results regarding the nutrient content of organic foods compared to conventional foods. Some evidence suggests differences in organic fruits, vegetables, and cereals. The most consistent is the increase in phytochemicals with antioxidant activity in organic crops, mostly phenolic chemicals [5,21,24,25]. Higher vitamin content, such as vitamin C, carotens/carotenoids, tocopherol, and folate, has also been observed [5,24,25]. Similarly, increments in zinc, manganese, calcium, potassium, phosphorus, magnesium, and iron have been reported [5,24,25]. Higher levels of omega-3 fatty acids have been obtained in organic dairy products, as well as iodine and selenium [5,26]. Organic meat presents a better fatty acid profile than conventional meat, with a higher content of PUFA and omega-3 [5,26,27]. On the contrary, studies show lower levels of proteins, nitrate, and nitrite in organic foods [5,24].

Nevertheless, controversy still exists. Some studies failed to show nutritional differences, and the nutritional significance of the changes is still unknown [19]. Recently, the European Food and Safety Authority (EFSA) considered that “a cause and effect relationship cannot be established between the consumption of organic foods and contribution to the protection of body cells and molecules (lipids and DNA) from oxidative damage” [28].

All this controversy over the nutritional differences between organic and conventional foods is mostly about natural foods. Regarding processed foods, only a few studies have been performed in Italy, Greece, and the USA [29,30,31,32,33,34,35,36]. Some of them used a small sample for the organic alternatives or very heterogeneous food groups, which limited their conclusions [31,33,35,36]. In addition, the question of whether the changes observed in organic foods were sufficient to make them healthy was not addressed in any of those works. Only two other publications applied any criteria to determine the healthiness of organic food but did not study the nutrient composition [29,36]. Therefore, a complete study of organic foods as well as a comparative analysis with conventional foods in terms of both nutrient composition and healthiness has not been published.

The use of NCs to promote organic foods has only been briefly reported in one study, though no statistical analysis was performed to compare with conventional foods [31]. As for nutrient fortification, no publication has yet been released.

Therefore, this is the first comprehensive study of significant samples of six specific organic food types, analysing their nutritional quality as well as their use of NCs and fortification. In parallel, a comparison with conventional food is also carried out. The assessment of the nutritional quality follows a two-way approach: the analysis of the nutrient composition and the application of a nutrient profile model (NPM) to determine their “healthiness”.

## 2. Materials and Methods

### 2.1. BADALI Database of Food Products Available in the Spanish Market

The data used in this work come from the Food Database, BADALI, developed at Miguel Hernández University [37]. This database was initially conceived as a social project to improve citizens diets and can be freely accessed online at the BADALI web page [37]. It includes information about foods available in the Spanish market, such as nutrient composition, some ingredients, and health and nutrition claims. Most of the items included at BADALI are processed since fresh foods are exempt from the mandatory nutrition declaration in Europe according to Annex V of Regulation (EC) No. 1169/2011 [38]. The inclusion criteria were: (1) foods sold in any Spanish online supermarket; and (2) foods with a nutrient declaration. Initially, brands were chosen according to information availability following the methodology described in Ropero et al., 2020 [39]. Manufacturers’ web pages were preferentially used, although when they were not available or information was missing, online supermarkets’ web pages were also acceptable (either the information transcribed into the web page or the food images provided). As a general rule, fewer nutrients were displayed on the retailers’ online web pages.

The information was extracted and reviewed by the researchers, and inconsistent data was not used for further analysis. In addition, the database was checked for duplicates according to the nutrient composition, and these were removed.

For the present study, data was collected from 2021 to 2023. Images of the package and information available on the manufacturer’s/supermarket’s web page were checked for the presence of any of these words: eco, ecologic (ecológico in Spanish), bio, and organic (orgánico in Spanish). Related symbols were also accepted. A previous version of the entire database (foods collected from 2017 to 2022) was used to select the food types with a high prevalence of organic alternatives. Then the sample was increased specifically for those food types. Table S1 shows the description of those finally included in this work.

### 2.2. Nutrient Composition Analysis and Evaluation of the “Healthiness”

Statistics were applied to determine differences in nutrient composition between organic and conventional foods (see Section 2.5). However, to consider statistically significant divergences as nutritionally relevant, the definition of the NCs as “increased” or “reduced” included in Regulation (EC) No 1924/2006 was used [40]. To consider that organic foods present an increased content in any of the nutrients analysed, the median has to meet the criteria ‘source of’ and increase by at least 30% compared to the median for conventional foods [40]. The definitions of “source of proteins” and “source of fibre” included in the regulation were followed, and the median for the energy was used when required. The definition of ‘source of’ for the rest of the nutrients is not included in the regulation. Therefore, only the criteria of at least a 30% increase was used. To consider that organic foods present a reduced content in any of the nutrients analysed, the reduction in the median has to be at least 30% compared to the conventional, except for 25% for sodium [40].

The Pan American Health Organization Nutrient Profile Model (PAHO-NPM) was used to classify foods as “healthy” or “less healthy” following previous works [41,42,43,44]. Foods were considered “less healthy” when they exceeded the thresholds for any of the critical nutrients (sodium, free sugar, total fat, saturated fat, trans-fat), or contained any low- and no-calorie sweeteners (LNCS) [41]. The criteria for trans-fat could not be applied because none of the products provided the content. This criteria was previously used to

The thresholds used were as follows: (1) ≥1 mg sodium/kcal; (2) ≥10% of total energy from free sugars; (3) ≥30% of total energy from total fat; and (4) ≥10% of total energy from saturated fat [41]. Only foods with data for all five components (sodium/salt, sugar, total fat, saturated fat, LNCS) were included in the global statistics for “less healthy”.

Free sugars on cereal-based products were estimated following the methodology already published in Beltrá et al., 2020 [44]: (1) For cereal products with no added sugar, the percentage of energy as free sugar was 0; (2) 2 g sugar/100 g was subtracted from total sugar for all cereal derivatives because this is the naturally occurring sugar content in most frequently used grains [45]. For dairy substitutes (not included in ref [44]), we followed the definition published by Public Health England in 2018: all sugars in milk, yoghurts, and dairy dessert substitutes were considered free, including those added as puréed fruit [46].

As mentioned above, the presence of LNCS in foods was registered as part of the PAHO-NPM. LNCS analysed here were both no-calorie as well as polyols (low-calorie). Some LNCS have technological functionalities in food products other than acting as sweeteners. This is the case with polyols (sorbitol, maltitol, isomalt, etc.). In this work, the presence of LNCS was recorded regardless of their function in the product.

Although the PAHO-NPM was not intended for use with unprocessed or minimally processed foods, we applied it to the entire sample because this is a research project.

### 2.3. Nutrition Claims (NCs) Analysis

Nutrition claims (NCs) were analysed following previous publications [39,47]. Information provided on the online supermarkets’ web pages was very limited regarding these claims. Therefore, in order to avoid any bias, only the main food image provided was checked for NCs. Six products were excluded because clear images could not be obtained.

Only NCs displayed as text were considered for the analysis. Some pictorial, graphic, or symbolic representations may be interpreted as NCs. However, though included in Regulation No. 1924/2006 [40], they were uncommon and could easily be misinterpreted. Therefore, they were not considered for the analysis. According to this regulation, only NCs listed in the annex are permitted [40]. These authorised NCs were grouped into categories for easier analysis according to the food component claimed (energy or nutrient). “Other” included all NCs about nutrients that were not specifically stated in this classification. NCs not listed in the annex, either specific or general (such as “nutritious” or “no sucrose added”), were considered not-authorised and classified as “Other”.

Some NCs were associated with health claims (example: “it contains iron, which contributes to the reduction of tiredness and fatigue”). In this case, they were listed as NCs as long as the condition required to use the health claim was a NC (in the example, the condition is to be “source of”, which is a NC) [48].

When required, some extra criteria for the analysis of NCs were used:

- NCs of the types “with/high minerals”, “with/high vitamins”, and “with/high vitamin B” were considered one NC.
- NCs of the type “with 7 vitamins” were registered as seven NCs.

### 2.4. Fortification with Vitamins and Minerals

The inclusion criteria to consider that a product is fortified with vitamins or minerals were as follows: (1) a chemical providing a vitamin/mineral must be listed as an ingredient, and (2) there must be no indication of an additive function for this chemical. When the same compound provided two minerals, it was acknowledged that the product was fortified with both. As an example, when calcium phosphate was added, it was listed as fortified with both calcium and phosphorus. The algae *Lithotammium Calcareum* was considered a means of fortifying with calcium.

### 2.5. Statistics

The Kruskal-Wallis H test (sometimes also called the “one-way ANOVA on ranks”) is a rank-based nonparametric test that can be used to determine statistically significant differences between two or more food groups of an independent variable on a continuous or ordinal dependent variable. Nonparametric ANOVA has no assumption of the normality of random error, but the independence of random error is required. The chi-square test of homogeneity was used to determine whether different columns (or rows) of data in a table come from the same population or not (i.e., whether the differences are consistent with being explained by sampling error alone). The significance level was set at *p* < 0.05 in all statistical analyses.

The statistical analysis of the application data in this work was performed with Microsoft Excel and Google Colab with Jupyter Notebooks and the libraries scikit-learn 0.22.2.post1, Pandas v0.25.3, and Matplotlib Python v3.2.0.

A principal component analysis (PCA) with varimax rotation was executed to reduce the dimensionality of the dataset, increase interpretability, and, at the same time, minimise information loss. We use the library scikit-learn 1.2.1 to preprocess, normalise, and calculate the principal components.

## 3. Results

### 3.1. Description of the Sample and Nutrient Composition

A total of 1886 processed foods were used for this study, 42.6% of them organic. They were classified into six specific food types (Table 1 and Table S1). The sample was large for all food types, as well as the conventional and organic categories, which allowed a meaningful statistical analysis.

The nutrient composition of organic foods is shown in Table 2, and some results may be highlighted. As expected, all cereal-based food types presented a high content of carbohydrates. Organic biscuits had high levels of sugar, total and saturated fat, as well as energy. Organic breakfast cereals presented medium sugar content, and sodium was particularly high in toasted bread and similar foods. Sodium was also high in biscuits and cereal cakes/crackers. As for dairy substitutes (milk, yoghurt, and desserts), carbohydrates were the only nutrient with a significant content, while the rest were minor.

When the nutrient content of organic foods was compared to conventional foods, some divergences were observed, although they depended on the food type. Biscuits presented nutritionally significant differences only in saturated fat and sodium, both lower for the organic version (41% and 32%, respectively) (Table 2). The decrease in sugar content was poor (12.5%). The proportion of conventional biscuits containing low- and no-calorie sweeteners (LNCS) was higher than that of organic biscuits (Table 3), which may be masking stronger differences. When these biscuits were excluded, the sugar decrease in the organic alternative was higher, yet not nutritionally relevant (19%) (Table S3).

Organic toasted bread and similar products did not present nutritionally relevant differences (Table 2). Important reductions in sugar and sodium content were observed in organic breakfast cereals (47% and 93%, respectively) (Table 2). Since these nutrients are low in the original grains, the high values were the result of added sugar and sodium. In this regard, we observed that an important proportion of these foods were elaborated exclusively with cereals, with no other ingredients (“only cereals” subtype, Tables S1 and S2). The prevalence was 3-fold higher in the organic than in the conventional (Table S2). When they were removed from the analysis (“more ingredients”, Tables S1 and S2), the divergences in sugar became nutritionally irrelevant (21% decrease) (Table S3). However, the sodium difference persisted, though reduced (68%; Table S3).

For cereal cakes/crackers, nutritionally significant differences were obtained for total fat, sodium, and sugar, all of which were lower for the organic version (80%, 43%, and 66%, respectively). We observed that some foods in this category had chocolate, which may account for some of these differences. In fact, as many as 41% of conventional cereal cakes/crackers included cacao/chocolate as an ingredient, while only 17.1% were organic (Table S2). When these were removed, total fat values were practically the same, the difference in sugar became nutritionally irrelevant, and that for sodium persisted (Table S3).

Organic milk substitutes only presented a nutritionally significant increase in carbohydrate (31%; Table 2). Despite the 27% decrease in proteins, values were already low in the conventional foods. As shown in Table S1, these drinks were elaborated with a diversity of ingredients (cereals, coco, nuts, soy). High carbohydrate cereals (rice and oats) were the main ingredient in 56.9% of organic drinks and 33.3% of conventional drinks, which may account for the higher values of this nutrient in the organic. As for yoghurts/dairy dessert substitutes, no nutritionally relevant differences were obtained (Table 2).

The comparison in the nutritional profile between conventional and organic food was next described by two principal components (PCs), by food type (Figure S1). The main advantage of this analysis is that the complexity of a comprehensive comparison based on eight nutrients and energy is simplified into two components (PC1 and PC2). The relative importance of each nutrient is calculated, and the result is a two-dimensional plot where global differences may be visualised as two separate dispersion groups for organic and conventional. These two PCs explained 69.4% of the total variability among products, with 44% and 25.3% for PC1 and PC2, respectively. According to this analysis, organic and conventional run undistinguishable as two sets of foods with similar nutritional properties.

### 3.2. Nutrient Profile Model

The results described above show a slight improvement in the nutritional quality of the organic foods studied compared to conventional foods. However, the question remains whether this is sufficient to consider organic food healthy. To address this, the use of a nutrient profile model (NPM) or a similar tool is required. In this work, we applied the Pan American Health Organization Nutrient Profile Model (PAHO-NPM) [41]. Foods critical in free sugar, total fat, saturated fat, sodium, or with any low- and no-calorie sweeteners (LNCS) are considered “less healthy” by the PAHO-NPM (trans fat could be evaluated because of a lack of data).

According to the PAHO-NPM, as many as 80.7% of organic foods were “less healthy” (Table 3). Free sugar is the condition with the highest percentage of organic foods exceeding the threshold (59%), followed by total fat (40.6%) (Table 3). On the contrary, only two organic foods contained LNCS (Table 3).

Compared to conventional foods, organic foods presented a lower prevalence of “less healthy” foods (14.5% less) (Table 3). The food types with the strongest divergence were breakfast cereals and cereal cakes/crackers (35% and 50% less, respectively, for organic) (Table 3). The difference greatly diminished when breakfast cereals containing only grain were excluded (the “more ingredients” subtype) (a 12% reduction) (Table S4).

When looking at individual nutrients, the result varied depending on the food type (Table 3 and Table S4). The prevalence of foods high in any of the critical nutrients for cereal cakes/crackers was lower for the organic version, except for LNCS. However, they all vanished when the cacao/chocolate-free items were excluded, except for sodium (Table S4). This result indicates that the differences were mostly due to a higher percentage of foods with cacao/chocolate among the conventional (Table S2). As for breakfast cereals, the organics presented a lower percentage of foods high in free sugar (41%; Table 3). When foods elaborated only with cereals were excluded (“more ingredients” subtype), this difference greatly diminished (17% decrease in the organic, Table S4). This suggests that most of the divergence was due to the higher presence of the “only cereal” subtype among the organics (Table S2). The other difference was the absence of any organic breakfast cereal with LNCS (Table 3).

Organic biscuits high in saturated fat, sodium, and LNCS were less than conventional biscuits (33%, 74%, and 90%, respectively) (Table 3). Yoghurts/dairy dessert substitutes diverged only in sodium content (90% lower for organic), even though there were no differences in sodium content. This may be due to the higher energy content of the organic version, which allows a higher tolerance for sodium (the threshold is 1 mg/kcal) (Table 2). The prevalence of foods high in fat was lower in organic milk substitutes than in conventional (36%) (Table 3). Another difference was the total absence of LNCS in the organic version. Finally, toasted bread and similar foods did not present differences in any nutrient (Table 3).

### 3.3. Nutrition Claims (NCs)

**Figure 1:**
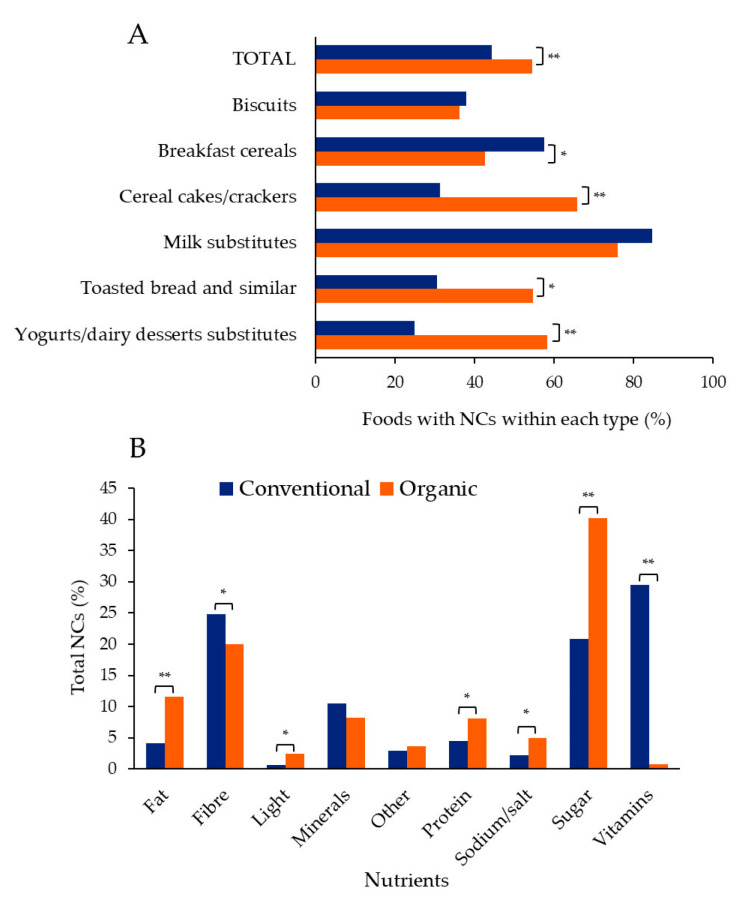
The presence of nutrition claims (NCs) in conventional and organic foods. (**A**) Percentage of foods with NCs within the food type. (**B**) Percentage of total NCs, by nutrient. Statistically significant differences according to *p* < 0.05: * *p* < 0.05; ** *p* < 0.001. The data plotted can be consulted in detail in Tables S5 and S6.

We next studied the use of NCs in organic foods. As shown in Figure 1 and Table S5, as many as 54.6% of organic foods made NCs. The food types with the highest prevalence were milk substitutes (76%), followed by cereal crackers/cakes (65.9%) (Figure 1 and Table S5). On the contrary, only 36.2% of biscuits used NCs on the food image (Figure 1 and Table S5).

When compared, organic foods presented a higher prevalence of NCs than conventional foods (23% more), although it depended on the food type (Figure 1, Table S5). Four of the six food types displayed differences (Figure 1, Table S5). While organic cereal cakes/crackers, toasted bread and similar products, and yoghurts/dairy dessert substitutes had higher prevalence than conventional ones, the opposite was the case for breakfast cereals (Figure 1 and Table S5).

Individual NCs were next analysed, and a total of 1551 were detected in the main food image (see Material and Methods for details) (Table S6). Vitamins were the most frequently claimed nutrients for conventional food, while sugar was for organic food (Figure 1 and Table S6). In fact, the NCs for sugar were double in organic foods, and only five NCs were for vitamins (<1%). Furthermore, the proportion of NCs dedicated to seven of the nine specific nutrients was statistically different (Figure 1 and Table S6). Therefore, organic and conventional food presented completely different NC profiles.

### 3.4. Fortification with Vitamins and Minerals

As mentioned in the introduction, nutrient fortification may be more appealing to consumers. Therefore, we next studied the micronutrient fortification of organic foods. The results were startling. Only two organic foods were fortified with vitamins (0.3%), while the prevalence was 18.4% among conventional foods (Table 4). As for minerals, the fortification of conventional food was more than 3-fold higher than that of organic food (Table 4). Curiously, all but one organic food fortified with minerals were milk substitutes, all of them with calcium.

## 4. Discussion

The main conclusion of this work is that organic food products are not as healthy as generally considered. Despite their high use of NCs and a slightly improved nutrition quality compared to conventional foods, most organic foods are classified as “less healthy” according to the PAHO-NPM [41].

Organic foods present an improved nutrient profile compared to conventional foods. However, many of the differences, though statistically significant, are not nutritionally relevant. In addition, some of the divergences observed were due to the existence of some heterogeneity among some food types. This is the case for organic breakfast cereals, with a higher proportion of the healthier subtype “only cereals” (with only grains and no other added ingredients). Similarly, conventional cereal cakes/crackers presented a higher proportion of those with cacao/chocolate, which increased total fat and sugar.

Surprisingly, the micronutrient fortification rate was much lower for organic foods than for conventional foods.

### 4.1. Nutrient Composition and Nutritional Quality

Our results show that most organic food products are “less healthy” according to the PAHO-NPM (81%) [41]. “Less healthy” foods are undoubtedly major contributors to unhealthy diets. The World Health Organization (WHO) considers unhealthy diets to be one of the main risk factors for chronic diseases (noncommunicable diseases, NCDs), along with tobacco use, physical inactivity, and the harmful use of alcohol [49]. The same institution acknowledges: “NCDs kill 41 million people each year, equivalent to 74% of all deaths” [49]. Therefore, the high percentage of “less healthy” organic food does not help to alleviate this burden of disease.

More than half of the organic products analysed (59%) exceeded the threshold and were considered high in free sugar. Already in 2015, WHO stated that “a high level of free sugars intake is of concern, because of its association with poor dietary quality, obesity and risk of NCDs” [50]. Recently, the European Food and Safety Authority (EFSA) declared that “the intake of added and free sugars should be as low as possible in the context of a nutritionally adequate diet” [51]. Free sugar intake in adults in Europe “ranges from about 7–8% of total energy intake in countries like Hungary and Norway to 16–17% in countries like Spain and the United Kingdom” [52]. Given this situation, a positive contribution of organic foods to reversing the high free sugar intake is much desired. However, the data shown in this work suggests otherwise.

As many as 40.6% and 19.4% of the organic foods analysed were high in total and saturated fat, respectively. Two food types presented important rates of organic foods high in saturated fat: biscuits (40.9%) and yoghurts/dairy dessert substitutes (32.5%). According to the EFSA, “the main dietary determinant of blood LDL cholesterol concentrations is saturated intake” [53]. The WHO guidelines draft launched in 2018 stated: “meta-analysis of randomised controlled trials conducted in adults found that reducing saturated fatty acid intake reduced the risk of cardiovascular events” [54]. Therefore, the significant rates of specific organic foods high in saturated fat do not contribute to lowering the intake of this nutrient.

Regarding sodium, as many as 83% of organic toasted bread and similar foods were high in sodium. Processed food accounts for around 75% of the total sodium intake in Europe and North America [55]. This greatly contributes to a global intake of twice the recommended amount [56]. The need to follow the guidelines arises from the fact that high sodium intake is a risk factor for NCDs and is associated with high blood pressure, coronary heart disease, and stroke [57]. In fact, one of the best investments to diminish NCDs is to reduce sodium intake [58].

Therefore, consumers’ perceptions that organic food products are healthy from a nutritional point of view are unfounded. The high presence of nutrients negatively associated with health in many organic food products is an important rising risk factor.

When the nutrient composition of organic foods was compared to conventional foods, the most consistent and important difference observed was a strong reduction in sodium values in three cereal-based food types (a 32–93% decrease). In addition, the proportion of organic foods high in sodium according to the PAHO-NPM was substantially lower in two of those food types (biscuits and cereal cakes/crackers; 74% and 47%, respectively). A study in Italy followed a similar approach but used the World Health Organization Sodium Benchmarks [36]. The authors did not apply statistics to determine differences between organic and conventional foods, but an important reduction in the proportion of foods above the benchmark could be deducted for organic cracker/savory biscuits [36]. This food type is similar to our organic cereal cakes/crackers, which also displayed a lower proportion of foods high in sodium.

This decrease in sodium content in some organic foods is in line with the results of a study performed with Polish costumers. Authors showed that those purchasing organic food attached high importance to salt (sodium) content information [59].

Some previous works have been published comparing the nutrient profiles of organic vs. conventional foods in Italy, Greece, and the USA [29,30,31,32,33,34,35,36]. However, the sample used for the organic version was rather small, or the food groups were very heterogeneous in some of them [31,33,35,36]. In the present study, an effort was made to reduce heterogeneity in food types. Importantly, the sample was significant for all six food types and for both conventional and organic (from 53 to 476 items/condition).

Our results are in line with a work published in 2021 in the USA with a large sample. Authors concluded that organically packaged foods have a more healthful profile than conventional [32]. They found that organic food had lower total sugar, added sugar, saturated fat, and sodium content [32]. However, they did not study the healthiness of the products.

The data presented here on milk substitutes (plant-based drinks) is quite similar to the results obtained in Italy by Angelino et al. [30]. They observed increases in energy and carbohydrates and a reduction in protein in the organic version [30].

Regarding cereal-based foods, Dall’Asta et al. did not find differences for sweet cereal-based foods or bread and substitutes [31]. On the contrary, we observed some differences in biscuits and breakfast cereals.

Some studies have investigated other food types. Lower energy, total fat, sugars, fibre, and salt were described in organic meat analogues in Italy [34]. Small differences were observed in organic compared to conventional pasta in another Italian work (7% increase in fibre; 14% decrease in proteins) [33].

Some studies have reported that consumers believe organic food to be lower in fat and calorie content as well as higher in fibre [8,10,11]. We obtained lower fat content only in organic cereal cakes/crackers because many of the conventional ones had cacao/chocolate. We did not observe any nutritionally relevant reduction in energy or fibre. Therefore, according to our results, consumers’ perceptions about organic products, specifically in terms of fat, energy, and fibre, are wrong.

The nutritional improvements observed in the present work usually correlate with a better PAHO-NPM score. Organic breakfast cereals were an exception because they contained much lower levels of sodium (93% less), though no changes in the proportion of foods high in sodium were obtained. They only scored better for free sugar, which correlates with an important reduction in total sugar compared to conventional. This result partly agrees with a ten-year-old study performed on ready-to-eat breakfast cereals in the USA [29]. Using the NuVal score, based on 19 nutritional attributes, the authors did not observe differences between conventional and organic [29]. The correlation between the nutrient composition results and the PAHO-NPM score also failed for milk substitutes. In this case, the organic version presented a lower proportion of foods high in fat, while no nutritionally relevant differences were observed for total fat content. Similarly, yoghurts/dairy dessert substitutes diverged in the percentage of those high in sodium (lower for organic), even though there were no differences in sodium content. As stated above, this may be due to the higher energy content of the organic version, which allowed a higher tolerance for sodium.

Finally, it is remarkable that the organic foods analysed in the present work rarely used LNCS. The purpose may well be to prevent any misgivings by consumers. Organic foods are considered healthy, while consumers’ perceptions of LNCS are not generally positive [6,7,8,60,61]. The use of LNCS may deteriorate this good opinion of organic foods.

### 4.2. Nutrition Claims in Organic Foods

Our results show that organic foods use NCs very frequently, even more than conventional foods, yet it depends on the food type. To the best of our knowledge, the only work published so far studying the use of NCs in organic foods and comparing them with conventional is one by Dall’Asta in 2020 [31]. Although they applied statistics, the number of items with an NC was relatively similar for all categories of organic and their conventional counterparts. The only exception was organic “pasta, rice, and other cereals”, with a higher prevalence [31]. Their results and ours disagree.

As mentioned in the Introduction, nutrition and health claims may increase the perceived nutritional quality of products and their healthiness, as well as influence purchasing behaviour [14,15,16,17]. This may be of major importance to organic consumers, particularly because those with a high health attitude attribute high importance to NCs [18]. The use of NCs by manufacturers may add to the general perception that organic food is healthy when, in fact, it is not.

Interestingly, organic and conventional food presented completely different NC profiles. While organic focused on sugar and fibre, conventional foods preferentially used vitamins. NCs related to sugar content can influence the perceived healthfulness of products, making them seem healthier than they are, and thus influencing food purchase intentions [17]. However, it may be misleading because, according to our results, as many as 59% of organic foods were high in free sugar.

The few NCs about sodium/salt in the organic products are somehow surprising, since this is the nutrient with the highest reductions. A possible explanation is that the knowledge, attitudes, and behaviours related to dietary salt intake are generally low in high-income countries [62]. According to a systematic review of 24 studies across 12 countries, consumers are aware of the health implications of a high salt intake. However, fundamental knowledge regarding recommended dietary intake, primary food sources, and the relationship between salt and sodium is lacking [62].

### 4.3. Fortification with Vitamins and Minerals

To our knowledge, there are no reports on the fortification of organic food products with vitamins and minerals. According to our data, these are poorly fortified, which correlates well with the low prevalence of NCs about these micronutrients, particularly vitamins.

Although some organic vegetable drinks have added calcium, they do so in a low proportion. As for organic yoghurt/dessert substitutes, none of them were fortified with minerals. Regarding vitamins, only one of all the organic dairy substitutes was fortified. This is particularly surprising because these plant-based drinks, desserts, and fermented drinks are usually presented as dairy substitutes. For this purpose, they are usually fortified with calcium and with vitamins that consumers usually associate with milk, such as vitamin A, D, and B12. This lack of fortification is detrimental to this dairy substitution claim.

### 4.4. Strengths and Limitations

The present work has some important strengths:

- This is the first comprehensive study of organic foods, analysing their nutritional quality following a two-way approach: the analysis of the nutrient composition and the application of a NPM to determine their “healthiness”;
- This is the first paper published studying the differences between organic and conventional food products in the Spanish market;
- This is the first paper describing in some detail the use of NCs in organic foods and comparing them with conventional foods;
- This is the first paper describing the fortification of organic and conventional food products with micronutrients;
- The food types studied were selected for the high prevalence of the organic version among those included in the Spanish food database, BADALI;
- The sample per food type and condition (conventional and organic) is significant;
- Data were collected several years after Regulation (EC) No. 1924/2006 on nutrition claims was fully in force [40].

Our work has a few important limitations:

- The selection of brands did not follow criteria based on customers’ purchases or the most popular products;
- The data collected was dependent on the accuracy of the information provided on the manufacturers’ and supermarkets’ web pages;
- The sample studied may not be representative of the extensive Spanish market for the food types analysed;
- Some of the products displayed 0 g of salt/sodium, which could be wrongly rounded. The EC published a guidance document with rounding instructions, but it is not compulsory [63].
- Only the main food image was used for the study on the use of NCs in order to preserve rigour throughout the sample.

## 5. Conclusions

The main conclusion of this work is that consumers’ perception that organic food products are healthy is unfounded from a nutritional point of view. The high use of NCs by manufacturers may add to this general belief. However, our results show that most organic food products are considered “less healthy”. The high presence of nutrients negatively associated with health in many organic food products is an important rising risk factor. The worst of all is that consumers are not aware of this.

## Figures and Tables

**Table 1:** Items included in the study and distribution by conventional and organic food types.

Food Types	No Foods		
	Total (%) *	Conventional (%)	Organic (%)
Biscuits	640 (33.9) *	476 (74.4)	164 (25.6)
Breakfast cereals	424 (22.5) *	207 (48.8)	217 (51.2)
Cereal cakes/crackers	165 (8.7) *	83 (50.3)	82 (49.7)
Milk substitutes (plant-based drinks)	315 (16.7) *	111 (35.2)	204 (64.8)
Toasted bread and similar	198 (10.5) *	145 (73.2)	53 (26.8)
Yoghurts/dairy dessert substitutes	144 (7.6) *	60 (41.7)	84 (58.3)
Total	1886 (100) *	1082 (57.4)	804 (42.6)

%: percentage within the food type; %*: percentage of the total sample.

**Table 2:** Energy and nutrient density of specific food types. Values in 100 g or 100 mL.

**Food Types**	**Organic**	**Energy (kcal)**			**Protein (g)**			**Carbohydrates (g)**			**Sugar (g)**		
		***n***	**Median (IR)**	***p*** **-Value**	***n***	**Median (IR)**	***p*** **-Value**	***n***	**Median (IR)**	***p*** **-Value**	***n***	**Median (IR)**	***p*** **-Value**
Biscuits	No	476	472 (449; 497)	0.487	476	6.4 (5.6; 7.2)	<0.001 *	476	66 (62; 70)	<0.001 *	473	24 (19; 33)	<0.001 *
	Yes	164	475 (455; 491)		164	7 (6.1; 8.2)		164	63 (60; 67.9)		161	21 (17; 25)	
Breakfast cereals	No	207	388 (374; 423)	<0.001 *	207	8.9 (7.4; 11.8)	<0.001 *	207	67 (61; 77)	<0.05 *	207	18 (10.3; 24.8)	<0.001 *
	Yes	209	376 (361; 405)		217	10 (8.9; 12)		209	64.3 (59.4; 72)		213	9.5 (1.2; 19)	
Cereal cakes/crackers	No	83	431 (382; 468)	<0.001 *	83	7.2 (6.9; 8.3)	<0.01 *	83	71 (67.3; 79.8)	<0.05 *	82	3.4 (1; 24.3)	<0.01 *
	Yes	79	383 (375; 393)		82	8.3 (7; 9.5)		79	77.5 (72.5; 80.9)		82	1.2 (0.6; 4.2)	
Milk substitutes	No	111	43 (33; 57)	<0.05 *	109	1.1 (0.6; 2.2)	<0.05 *	111	6.1 (1.8; 9.2)	<0.001 *	110	4 (0.8; 5.9)	0.121
	Yes	200	48 (40; 57)		204	0.8 (0.5; 1.2)		200	8 (3.9; 10.5)		204	5 (2.4; 5.9)	
Toasted bread and similar	No	145	419 (394; 446)	0.081	144	11 (9.5; 12.5)	<0.001 *	144	66 (62; 71)	0.152	144	3.2 (1.9; 5.1)	<0.01 *
	Yes	53	411 (389; 423)		52	12.3 (11.4; 13.6)		53	67 (61; 69)		53	2.3 (1.4; 3.3)	
Yogurts/dairy desserts substitutes	No	60	82 (66; 88)	<0.01 *	60	3.4 (1.3; 3.7)	0.415	60	10 (5.4; 12.5)	0.073	60	7.9 (1.9; 10.6)	0.522
	Yes	84	90 (77;112)		84	2.2 (1; 3.8)		84	10.9 (5; 15.4)		84	8.4 (3.1; 11.3)	
**Food Types**	**Organic**	**Total Fat (g)**			**Saturated Fat (g)**			**Fibre (g)**			**Sodium (mg)**		
		***n***	**Median (IR)**	***p*** **-Value**	***n***	**Median (IR)**	***p*** **-Value**	***n***	**Median (IR)**	***p*** **-Value**	***n***	**Median (IR)**	***p*** **-Value**
Biscuits	No	476	20 (16; 24)	0.249	475	7.2 (2.2; 13)	<0.01 *	372	3.9 (2.5; 6)	0.233	476	262 (192; 349)	<0.001 *
	Yes	164	20.9 (16.7; 23)		164	4.3 (2.2.10)		144	4.6 (2.9; 6)		164	178 (110; 272)	
Breakfast cereals	No	207	6.6 (3.4; 12.6)	0.116	207	1.4 (0.8; 3)	<0.01 *	188	7 (4.9; 9.5)	0.332	206	164 (40; 300)	<0.001 *
	Yes	217	5.7 (2.5; 11)		213	1 (0.6; 2.1)		210	7.7 (5; 10)		211	12 (4; 86)	
Cereal cakes/crackers	No	83	14 (2.6; 17)	<0.001 *	83	1.7 (0.6; 10)	<0.01 *	71	3.5 (2; 4.6)	0.393	83	352 (76; 500)	<0.001 *
	Yes	81	2.8 (2.3; 3.8)		81	0.6 (0.5; 1)		71	3.4 (2.5; 4.6)		82	200 (40; 286)	
Milk substitutes	No	111	1.7 (1.1; 2)	0.249	111	0.2 (0.1; 0.3)	0.312	75	0.5 (0.3; 0.8)	0.526	111	40 (24; 48)	0.502
	Yes	204	1.4 (1; 2.1)		203	0.2 (0.2; 0.4)		129	0.5 (0.3; 0.8)		199	40 (32; 40)	
Toasted bread and similar	No	145	10 (6.6; 16)	0.242	145	1.7 (0.9; 2.9)	0.200	110	4.7 (4; 8.5)	0.258	145	600 (508; 720)	0.462
	Yes	53	9.7 (6; 12)		53	1.8 (0.7; 2)		49	6.3 (4; 8.8)		53	560 (476; 800)	
Yoghurts/dairy dessert substitutes	No	60	2.7 (2.1; 4.7)	0.177	60	0.4 (0.4; 1)	0.998	28	1 (0.7; 1.5)	ND	60	32 (12; 49)	0.374
	Yes	84	3 (2.2; 5.1)		83	0.4 (0.3; 2.5)		34	0.5 (0.4; 0.9)		84	40 (24; 40)	

*n*: Foods with data. IR: interquartile range. * Statistically significant differences according to *p* < 0.05. ND: not determined because of <30 foods/condition.

**Table 3:** Classification of foods as high in critical nutrients according to the Pan American Health Organization Nutrient Profile Model (PAHO-NPM) [41], by food type.

Food Types	Organic	Less Healthy			High Total Fat			High Free Sugar			High Saturated Fat			High Sodium			Sweeteners (LNCS)		
		*n*	No (%)	*p*-Value	*n*	No (%)	*p*-Value	*n*	No (%)	*p*-Value	*n*	No (%)	*p*-Value	*n*	No (%)	*p*-Value	*n*	No (%)	*p*-Value
Biscuits	No	472	472 (100)	<0.01 *	476	381 (80)	0.08	473	409 (86.5)	0.761	475	292 (61.5)	<0.001 *	476	55 (11.6)	<0.01 *	476	57 (12)	<0.001 *
	Yes	161	157 (97.5)		164	142 (86.6)		161	137 (85.1)		164	67 (40.9)		164	5 (3)		164	2 (1.2)	
Breakfast cereals	No	206	169 (82)	<0.001 *	207	40 (19.3)	0.173	207	146 (70.5)	<0.001 *	207	22 (10.6)	0.113	206	24 (11.7)	0.264	206	8 (3.9)	<0.05 *
	Yes	201	107 (53.2)		209	29 (13.9)		213	89 (41.8)		205	12 (5.9)		203	16 (7.9)		217	0 (0)	
Cereal cakes/crackers	No	83	71 (85.5)	<0.001 *	83	33 (39.8)	<0.001 *	83	31 (37.3)	<0.05 *	83	34 (41)	<0.01 *	83	36 (43.4)	<0.01 *	83	5 (6)	0.071
	Yes	79	34 (43)		79	11(13.9)		79	15 (19)		79	13 (16.5)		79	18 (22.8)		82	0 (0)	
Milk substitutes	No	111	111 (100)	1	111	70 (63.1)	<0.001 *	111	83 (74.8)	0.355	111	9 (8.1)	0.088	111	40 (36)	0.112	111	5 (4.5)	<0.01 *
	Yes	194	193 (99.5)		200	81 (40.5)		200	160 (80)		199	31 (15.6)		195	52 (26.7)		204	0 (0)	
Toasted bread and similar	No	144	134 (93.1)	0.778	145	40 (27.6)	0.103	144	4 (2.8)	0.512	145	14 (9.7)	0.294	145	128 (88.3)	0.464	145	1 (0.7)	1
	Yes	53	48 (90.6)		53	8 (15.1)		53	0 (0)		53	2 (3.8)		53	44 (83)		53	0 (0)	
Yoghurts/dairy dessert substitutes	No	60	59 (98.3)	0.87	60	34 (56.7)	0.977	60	47 (78.3)	1	60	16 (26.7)	0.569	60	7 (11.7)	<0.05 *	59	0 (0)	1
	Yes	83	83 (100)		84	49 (58.3)		84	65 (77.4)		83	27 (32.5)		84	1 (1.2)		82	0 (0)	
Total	No	1076	1016 (94.4)	<0.001 *	1082	598 (55.3)	<0.001 *	1078	720 (66.8)	<0.001 *	1081	387 (35.8)	<0.001 *	1081	290 (26.8)	<0.001 *	1080	76 (7)	<0.001 *
	Yes	771	622 (80.7)		789	320 (40.6)		790	466 (59)		783	152 (19.4)		778	136 (17.5)		802	2 (0.2)	

* Statistically significant differences according to *p* < 0.05. “Less healthy”: food exceeding any of the thresholds for critical nutrients or with sweeteners (LNCS). Thresholds used to consider foods high in critical nutrients [41] are ≥30% of total energy from total fat; ≥10% of total energy from free sugars; ≥10% of total energy from saturated fat; ≥1 mg sodium/kcal. *n*: Foods with data. No: Foods exceeding the threshold or with LNCS. LNCS = low- and no-calorie sweeteners.

**Table 4:** Foods with added minerals or vitamins, by food type.

Food Types	Organic	Foods with Added Minerals			Foods with Added Vitamins		
		*n*	No (%)	*p*-Value	*n*	No (%)	*p*-Value
Biscuits	No	476	40 (8.4)	<0.001 **	476	54 (11.3)	<0.001 **
	Yes	164	1 (0.6)		164	1 (0.6)	
Breakfast cereals	No	207	62 (30)	<0.001 **	207	65 (31.4)	<0.001 **
	Yes	216	0 (0)		216	0 (0)	
Cereal cakes/crackers	No	83	0 (0)	1	83	0 (0)	1
	Yes	82	0 (0)		82	0 (0)	
Milk substitutes	No	111	66 (59.5)	<0.001 **	111	66 (59.5)	<0.001 **
	Yes	203	42 (20.7)		203	1 (0.5)	
Toasted bread and similar	No	140	0 (0)	1	140	0 (0)	1
	Yes	53	0 (0)		53	0 (0)	
Yoghurts/dairy dessert substitutes	No	60	32 (53.3)	<0.001 **	60	13 (21.7)	<0.001 **
	Yes	82	0 (0)		82	0 (0)	
Total	No	1077	200 (18.6) *	<0.001 **	1077	198 (18.4)	<0.001 **
	Yes	800	43 (5.4) *		800	2 (0.3)	

*n*: Foods with data. No: Foods with added minerals/vitamins. %: percentage within the food type. *: percentage of the total. ** Statistically significant differences according to *p* < 0.05.

## Data Availability

Not applicable.

## Supplementary Materials

The following supporting information can be downloaded at: https://www.mdpi.com/article/10.3390/nu15081876/s1. Table S1: Description of the items included in the study, by food type and subtype; Table S2: Items included in the study and distribution in conventional and organic foods in specific subtypes; Table S3: Energy and nutrient density of specific food subtypes. Values in 100 g or 100 mL; Figure S1. Principal component analysis (PCA) based on the nutritional composition of products included in this study, by food type, for conventional and organic food. Nutrients considered (in 100 g or 100 mL): energy (kcal), proteins (g), carbohydrates (g), sugar (g), total fat (g), saturated fat (g), fibre (g), and sodium (g); Table S4: Classification of foods as high in critical nutrients according to the PAHO-NPM [41] of specific food subtypes; Table S5: Foods with nutrition claims (NCs), by food type; Table S6: Nutrition claims (NCs), by nutrient.
