# Supplementary material for: Nutritional Description of Organic and Conventional Food Products in Spain: The BADALI Project
Source: Nutrients. 2023 Apr 13;15(8):1876. doi: 10.3390/nu15081876 (PMC10144859; doi:10.3390/nu15081876)
Supplement: Supplementary file 1 [file nutrients-15-01876-s001.zip › nutrients-2323859-supplementary.pdf]

Table S1. Description of the items included in the study, by food type and subtype.

Food Types and Subtypes	Foods
Biscuits	All kinds of biscuits according to their commercial name, including waffers. Savoury biscuits were excluded.
Biscuits – LNCS free	Biscuits with no low- and no-calorie sweeteners added
Breakfast cereals	Flakes, muesli, granola, extruded, ready-to-eat cereals
Breakfast cereals – "only cereals"	Breakfast cereals elaborated only with cereals
Breakfast cereals – "more ingredients"	Breakfast cereals elaborated with cereals and additional ingredients
Cereal cakes/crackers	Cereal cakes and crackers with no yeast or gasifiers added
Cereal cakes/crackers – with cacao/chocolate	Cereal cakes/crackers with at least an ingredient adding cacao or chocolate
Cereal cakes/crackers – cacao/chocolate free	Cereal cakes/crackers free of any ingredient adding cacao or chocolate
Milk substitutes (plant-based drinks)	Vegetable drinks made of soya, oat, rice, coco, nuts, tigernut, canary seed or any other vegetable ingredient
Toasted bread and similar	Toasted bread and similar products made with yeast
Yogurts/dairy desserts substitutes	Fermented products and dairy desserts made of vegetable drinks

LNCS: low- and no-calorie sweeteners

Table S2. Items included in the study and distribution in conventional and organic in specific subtypes.

Food Types	Total (%)*	Conventional (%)	Organic (%)
Breakfast cereals – "only cereals"	97 (22.9)*	21 (10.1)	76 (35)
Breakfast cereals – "more ingredients"	327 (77.1)*	186 (89.9)	141 (65)
Cereal cakes/crackers – with cacao/chocolate	48 (29.1)*	34 (41)	14 (17.1)
Cereal cakes/crackers – cacao/chocolate free	117 (70.9)*	49 (59)	68 (82.9)

%: percentage within the conventional/organic version; %*: percentage of the total food subtype

Table S3. Energy and nutrient density of specific food subtypes. Values in 100 g or 100 mL.

Food subtypes	Organic	Energy (kcal)			Protein (g)			Carbohydrates (g)			Sugar (g)		
		**n**	**Median (IR)**	***p*-Value**	**n**	**Median (IR)**	***p*-Value**	**n**	**Median (IR)**	***p*-Value**	**n**	**Median (IR)**	***p*-Value**
Biscuits – LNCS free	No	419	475 (455; 500)	0.452	419	6.3 (5.5; 7)	< 0.001*	419	66 (62; 70)	< 0.001*	416	26 (21; 34)	< 0.001*
	Yes	162	476 (455; 492)		162	7 (6; 8.2)		162	63 (60; 67.8)		159	21 (18; 25)	
Breakfast cereals – "more ingredients"	No	186	390 (377; 425)	0.367	186	8.6 (7.2; 11)	< 0.05*	186	68.8 (63; 78)	< 0.05*	186	19.1 (13.2; 25)	< 0.01*
	Yes	138	388 (369; 431)		141	9.3 (8.1; 11)		138	65 (60.6; 73.8)		140	15 (9.8; 21.9)	
Cereal cakes/crackers – cacao/chocolate free	No	49	385 (379; 408)	< 0.05*	49	7.9 (7; 9)	< 0.05*	49	79 (72; 83)	0.823	48	1 (0.5; 3)	0.429
	Yes	66	381 (374; 388)		68	8.5 (7.6; 9.7)		66	78 (75; 81)		68	0.9 (0.5; 2.1)	

Food subtypes	Organic	Total fat (g)			Saturated fat (g)			Fibre (g)			Sodium (mg)		
		**n**	**Median (IR)**	***p*-Value**	**n**	**Median (IR)**	***p*-Value**	**n**	**Median (IR)**	***p*-Value**	**n**	**Median (IR)**	***p*-Value**
Biscuits – LNCS free	No	419	20 (16; 24)	0.357	418	7.7 (2.7; 13)	< 0.001*	318	3.5 (2.4; 5.5)	< 0.01*	419	272 (198; 351)	< 0.001*
	Yes	162	21 (16.8; 23)		162	4.3 (2.3; 10)		142	4.7 (2.9; 6)		162	178 (106; 272)	
Breakfast cereals – "more ingredients"	No	186	6.1 (3; 13.6)	0.654	186	1.7 (0.7; 3.3)	0.49	168	6.4 (4.7; 9.1)	0.285	185	200 (80; 308)	< 0.001*
	Yes	141	7.9 (2.6; 14.4)		140	1.6 (0.7; 3.3)		139	7.4 (4.8; 8.9)		136	64 (12; 154)	
Cereal cakes/crackers – cacao/chocolate free	No	49	2.6 (2; 8.5)	0.052	49	0.6 (0.3; 1.4)	0.462	42	2.7 (2; 4.2)	0.359	49	480 (352; 640)	< 0.001*
	Yes	68	2.7 (1.9; 3.2)		68	0.5 (0.5; 0.6)		59	3.2 (2.4; 4.5)		68	220 (112; 400)	

IR: interquartile range. * Statistically significant differences according to $p < 0.05$.

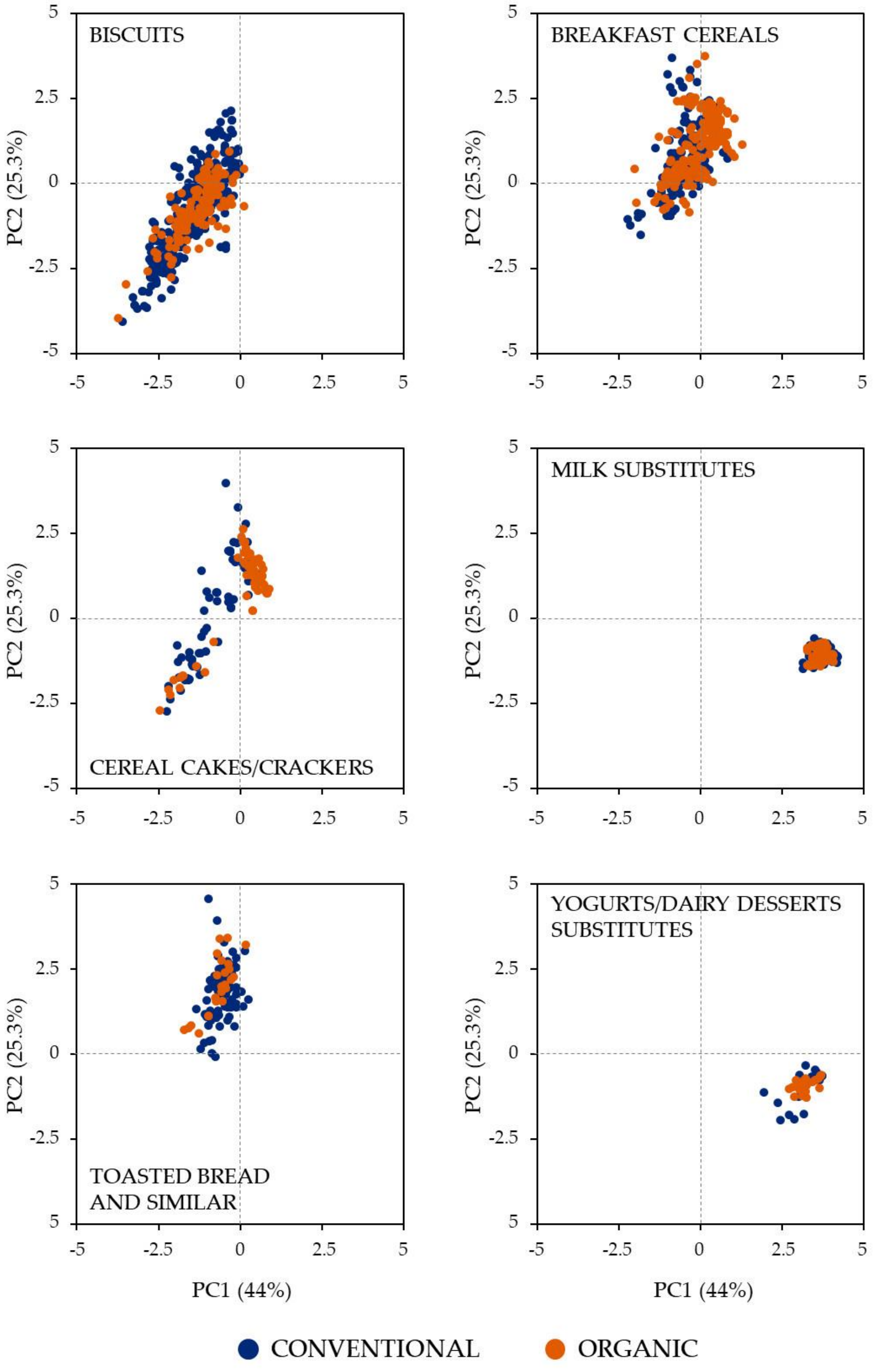

Figure S1. Principal Component Analysis (PCA) based on the nutritional composition of products included in this study, by food type, for conventional and organic food. Nutrients considered (in 100g or 100ml): energy (kcal), proteins (g), carbohydrates (g), sugar (g), total fat (g), saturated fat (g), fibre (g) and sodium (g).

Table S4. Classification of foods as high in critical nutrients according to the PAHO-NPM [41] of specific food subtypes.

Food subtypes	Organic	Less healthy			High total fat			High free sugar			High saturated fat			High sodium			Sweeteners (LNCS)		
		**n**	**No (%)**	***p*-value**	**n**	**No (%)**	***p*-value**	**n**	**No (%)**	***p*-value**	**n**	**No (%)**	***p*-value**	**n**	**No (%)**	***p*-value**	**n**	**No (%)**	***p*-value**
Biscuits – LNCS free	No	415	415 (100)	< 0.01*	419	337 (80.4)	0.08	416	408 (98.1)	< 0.001*	418	268 (64.1)	< 0.001*	419	48 (11.5)	< 0.01*	419	0 (0)	ND
	Yes	159	155 (97.5)		162	141 (87)		159	137 (86.2)		162	67 (41.4)		162	5 (3.1)		162	0 (0)	
Breakfast cereals –	No	185	169 (91.4)	< 0.01*	186	40 (21.5)	1	186	146 (78.5)	< 0.05*	186	22 (11.8)	0.481	185	24 (13)	0.937	185	8 (4.3)	< 0.05*
"more ingredients"	Yes	133	107 (80.5)		138	29 (21)		137	89 (65)		137	12 (8.8)		133	16 (12)		141	0 (0)	
Cereal cakes/crackers	No	49	37 (75.5)	< 0.001*	49	4 (8.2)	0.065	49	0 (0)	0.611	49	1 (2)	1	49	35 (71.4)	<0.001*	49	2 (4.1)	0.338
– cacao/chocolate free	Yes	66	21 (31.8)		66	0 (0)		66	2 (3)		66	1 (1.5)		66	18 (27.3)		68	0 (0)	

* Statistically significant differences according to $p < 0.05$. "Less healthy": food exceeding any of the thresholds for critical nutrients or with sweeteners (LNCS). Thresholds used to consider foods as high in critical nutrients [41]: ≥30% of total energy from total fat, ≥10% of total energy from free sugars, ≥10% of total energy from saturated fat, ≥1 mg sodium/kcal. n=Foods with data. No=Foods exceeding the threshold or with LNCS. LNCS=low- and no-clorie sweeteners. ND: not determined because the condition for this food subtype was the absence of LNCS.

Table S5. Foods with nutrition claims (NCs), by food type.

Food Types	No Foods with NCs (%)		*p*-value
	Conventional (%)	Organic (%)	
Biscuits	180 (37.9)	59 (36.2)	0.77
Breakfast cereals	118 (57.6)	92 (42.6)	< 0.01*
Cereal cakes/crackers	26 (31.3)	54 (65.9)	< 0.001*
Milk substitutes	94 (84.7)	155 (76)	0.095
Toasted bread and similar	44 (30.6)	29 (54.7)	< 0.01*
Yogurts/dairy desserts substitutes	15 (25)	49 (58.3)	< 0.001*
**Total**	477 (44.2)	438 (54.6)	< 0.001*

%: percentage within the food type. * Statistically significant differences according to p < 0.05. Data in this table is plotted in Figure 1A.

Table S6. Nutrition claims (NCs), by nutrient.

Nutrient	Total	No Foods with NCs (%)		*p*-value
		Conventional (%)	Organic (%)	
Fat	109	39 (4.1)	70 (11.6)	< 0.001*
Fibre	355	234 (24.8)	121 (20)	< 0.05*
Light	21	6 (0.6)	15 (2.5)	< 0.01*
Minerals	149	99 (10.5)	50 (8.3)	0.147
Other	50	28 (3)	22 (3.6)	0.468
Protein	91	42 (4.4)	49 (8.1)	< 0.01*
Sodium/salt	51	21 (2.2)	30 (5)	< 0.01*
Sugar	441	197 (20.8)	244 (40.3)	< 0.001*
Vitamins	284	279 (29.5)	5 (0.8)	< 0.001*
**Total**	1551	945	606	ND

%: percentage of total NCs in conventional or organic food. * Statistically significant differences according to p < 0.05. Data in this table is plotted in Figure 1B. ND: not determined.
